# Corpora Amylacea of Brain Tissue from Neurodegenerative Diseases Are Stained with Specific Antifungal Antibodies

**DOI:** 10.3389/fnins.2016.00086

**Published:** 2016-03-08

**Authors:** Diana Pisa, Ruth Alonso, Alberto Rábano, Luis Carrasco

**Affiliations:** ^1^Centro de Biología Molecular “Severo Ochoa,” Universidad Autónoma de MadridMadrid, Spain; ^2^Department of Neuropathology and Tissue Bank, Unidad de Investigación Proyecto Alzheimer, Fundación Centro de Investigación de Enfermedades Neurologicas, Instituto de Salud Carlos IIIMadrid, Spain

**Keywords:** corpora amylacea, neurodegenerative disease, fungal infection, Alzheimer's disease, amyotrophic lateral sclerosis

## Abstract

The origin and potential function of corpora amylacea (CA) remains largely unknown. Low numbers of CA are detected in the aging brain of normal individuals but they are abundant in the central nervous system of patients with neurodegenerative diseases. In the present study, we show that CA from patients diagnosed with Alzheimer's disease (AD) contain fungal proteins as detected by immunohistochemistry analyses. Accordingly, CA were labeled with different anti-fungal antibodies at the external surface, whereas the central portion composed of calcium salts contain less proteins. Detection of fungal proteins was achieved using a number of antibodies raised against different fungal species, which indicated cross-reactivity between the fungal proteins present in CA and the antibodies employed. Importantly, these antibodies do not immunoreact with cellular proteins. Additionally, CNS samples from patients diagnosed with amyotrophic lateral sclerosis (ALS) and Parkinson's disease (PD) also contained CA that were immunoreactive with a range of antifungal antibodies. However, CA were less abundant in ALS or PD patients as compared to CNS samples from AD. By contrast, CA from brain tissue of control subjects were almost devoid of fungal immunoreactivity. These observations are consistent with the concept that CA associate with fungal infections and may contribute to the elucidation of the origin of CA.

## Introduction

Corpora amylacea (CA) are glycoproteinaceous inclusions that accumulate in the brain during the course of normal aging and to a greater extent in some neurodegenerative diseases, particularly Alzheimer's disease (AD) (Mrak et al., 1997; Keller, 2006; Song et al., 2014). Abundant CA are found in a subset of patients with temporal epilepsy, where extensive deposits of CA replace the pyramidal layers of the cornu ammonis (Nishio et al., 2001; Kovacs and Risser, 2014). In addition to the central nervous system (CNS), CA are found in other organs and tissues, such as normal prostate glands, prostate cancer and several other malignant tissues (Christian et al., 2005; Morales et al., 2005; Hechtman et al., 2013a,b; Badea et al., 2015). CA are amorphous rounded, laminated bodies approximately 10-50 μm in diameter. The composition of CA has been analyzed in some detail. They mostly contain polyglucans (over 85% are hexoses) with a minor component (4%) of proteins (Robitaille et al., 1980; Nishimura et al., 2000; Sfanos et al., 2009). The rounded core is formed by different calcium salts, principally calcium phosphate and calcium oxalate depending on the bodies analyzed (Magura and Spector, 1979; Nakamura et al., 1995; Kodaka et al., 2008). A wide range of proteins are found in CA and a number of them have been characterized using specific antibodies (Singhrao et al., 1994). For example, ubiquitin, heat-shock proteins, Bcl-2, and c-Jun (Martin et al., 1991; Cisse et al., 1993; Botez and Rami, 2001) in addition to tau and several blood proteins such as thrombospondin-1 and some complement components may be detected in CA (Singhrao et al., 1993, 1995; Meng et al., 2009; Day et al., 2015). A detailed characterization of prostate CA by proteomic analyses has confirmed that lactoferrin is the most abundant protein, together with myeloperoxidase, S100 calcium-binding proteins A8 and A9, which form human calprotectin, and α-defensins, which form part of neutrophil granules (Sfanos et al., 2009). A number of S100 proteins including calprotectin, an inflammatory protein, are also present in CA from normal human brains (Hoyaux et al., 2000). Indeed, immunohistochemistry analysis suggests that the source of calprotectin in CA is prostate-infiltrating neutrophils, leading to the concept that chronic inflammation results in prostate cancer (De Marzo et al., 2007; Sfanos et al., 2009, 2014). By contrast, the suggestion that CA are built up of breakdrown products from neurons and oligodendroglial cells has also been proposed (Singhrao et al., 1994). Along this line, proteomic analyses of brain CA from multiple sclerosis patients detected the presence of cytoskeleton proteins and glycolysis enzymes (Selmaj et al., 2008). A number of microorganisms are suggested as the potential source of the chronic inflammation that triggers the formation of CA. Among these, several bacteria such as *Chlamydia trachomatis, Escherichia coli*, and *Pseudomonas* spp., protozoa such as *Trichomonas vaginalis* and viruses known to contribute to different types of cancer, including human papillomavirus, have been considered (Sfanos et al., 2009, 2014). Furthermore, a correlation between fungal infection and prostatic cancer has been reported (Sutcliffe et al., 2014). Prostatic CA are thought to give rise to prostatic calculi, and electron microscopy examination suggests the presence of microbial infection (Dessombz et al., 2012). Therefore, the traditional notion that CA result from precipitated proteins of glandular secretions is being replaced by the concept that they represent a response to a microbial infection.

We have recently reported the presence of fungal proteins in CNS from AD patients (Alonso et al., 2014a; Pisa et al., 2015a,b), and also in patients diagnosed with amyotrophic lateral sclerosis (ALS) (Alonso et al., 2015). Fungal infections elicit a neutrophil response, leading to the production of defensins and other molecules that participate in the innate immune response (Cunha et al., 2014; Lionakis, 2014). Neutrophils play a pivotal role against fungal infections (Lionakis, 2014). Undoubtedly, lactoferrin is considered a marker of inflammation and infiltration. Transferrin and lactoferrin are iron-binding proteins which function to maintain low levels of ferric ions in blood, mucus and tissues (Johnson and Wessling-Resnick, 2012). In this manner, some microbial infections are controlled since an increase in free iron leads to microbial growth (Samaranayake et al., 1997; Bullen et al., 2006; Mehra et al., 2012). Additionally, both myeloperoxidase and calprotectin are involved in the control of fungal infections (Murthy et al., 1993; Metzler et al., 2011). Finally, defensins are a family of small cationic peptides that can perturb the plasma membrane of *C. albicans*, leading to increased membrane permeability (Schroeder et al., 2011). As indicated above, polyglucans are the most abundant macromolecule in CA and interestingly these polysaccharides are also quite abundant in the fungal cell wall (Free, 2013). In the current study, we assessed the presence of fungal proteins in CA from different CNS regions obtained from control individuals and patients with several neurodegenerative diseases. Our findings provide strong evidence that fungal proteins are localized in the periphery of CA from patients diagnosed with neurodegenerative diseases.

## Materials and methods

### Description of control subjects and patients

We analyzed samples from patients diagnosed with AD, ALS, and Parkinson's disease (PD) in addition to control individuals without neurological disease. The age and gender of the subjects investigated in this study are listed in Supplementary Table 1. All samples were supplied by a brain bank (Banco de Tejidos CIEN) and were analyzed anonymously. The Ethics Committee of the Universidad Autónoma de Madrid approved the study. The transfer of samples was carried out according to national regulations concerning research on human biological samples. In all cases, written informed consent was obtained. For patients with dementia, informed consent for brain donation was given on a postmortem basis by a next-of-kin following the procedure established by the external ethical committee of the brain bank. Accordingly, a next-of-kin of the patient gave credit through an informed consent document to the fact that the patient had never opposed to be a brain donor during his/her life. An ethics committee external to the bank approved all ethico-legal documents, including written informed consent.

Brain samples were processed according to a common postmortem protocol followed by Banco de Tejidos CIEN. Briefly, rapid neuropathological autopsy was performed upon call by the donor's proxies (mean postmortem interval, 4.5 h). Immediately after extraction, the right half of the brain was sliced and frozen, while the left half was fixed by immersion in phosphate-buffered 4% formaldehyde for at least 3 weeks. A full neuropathological study was performed in the left half brain after fixation. Neuropathological diagnosis and staging of all disease entities was performed according to consensus criteria. Various neuropathological variables related to AD, vascular, Lewy and TDP (TAR DNA-binding protein) pathologies in addition to the presence of hippocampal sclerosis were recorded for full classification of cases.

### Antifungal antibodies

*Candida famata, C. albicans, C. glabrata*, and *Syncephalastrun racemosum* were grown in YEP (yeast extract peptone) medium (1% yeast extract, 2% Bacto peptone) as described (Pisa et al., 2008). Fungal cells were centrifuged and washed in phosphate-buffered saline (PBS). *Phoma betae* was purchased from Allergon AB (Engelholm, Sweden). Fungal cells were autoclaved and lyophilized. Rabbit antisera against *C. famata, C. albicans, C. glabrata, P. betae*, and *S. racemosum* were obtained by inoculation of 1 or 2 mg of dried fungi in 0.5 ml PBS, previously mixed with an equal volume of Freund's adjuvant. Rabbits were inoculated up to three times every 3 weeks and the antibody titer and specificity of the sera were tested by immunohistochemistry and immunoblotting using fungal proteins. The protocols employed were approved by the ethics committee of Centro de Biologia Molecular “Severo Ochoa” (identification number: ES280790000180). The optimal dilution for immunofluorescence staining for each antibody was assayed using both isolated *Candida* spp.

The specificity of the antifungal antibodies obtained was tested by immunofluorescence against different *Candida* spp. The cross-reactivity of each antibody against the different fungal species can differ, for instance anti-*C. glabrata* antibody immunoreacted with *C. glabrata, C. albicans, C. tropicalis, C. parapsilosis*, and *C. Krusei*, whereas anti-*C. albicans* does not recognize *C. Parapsilosis* and *C. Krusei*. By contrast, anti-*S. racemosum* only immunoreacts with *C. Krusei*. Besides, none of the antifungal antibodies obtained immunoreacted with cultured human cells or human brain sections from healthy subjects (Pacheco et al., 2007; Pisa et al., 2015a,b).

### Immunohistochemistry analysis

CNS tissue was embedded in paraffin following standard techniques and cut into 5-μ m sections using a microtome (Microm HM355s, Walldorf, Germany). For immunohistochemical analysis, paraffin was removed and sections were rehydrated and boiled for 2 min in 10 mM citrate buffer and then incubated for 10 min with 50 mM ammonium chloride. Subsequently, tissue sections were incubated for 10 min with PBS/Triton X-100 (0.1%) followed by 20 min with 2% bovine serum albumin in PBS. Sections were incubated overnight at 4°C with a mouse monoclonal antibody raised against human α-tubulin (Sigma), human phospho-PHF-tau, clone AT100 (Thermo Scientific), or human neurofilament protein, clone 2F11 (Dako), all at 1:50 dilution, or a rabbit polyclonal antibody raised against proteins obtained from *C. glabrata* at 1:500 or *C. famata, C. albicans, P. betae*, and *S. racemosum* at 1:100 dilution. Thereafter, sections were washed with PBS and further incubated for 1 h at 37°C with donkey anti-mouse IgG secondary antibody conjugated to Alexa 555 (Invitrogen) at 1:500 for α-tubulin, tau and neurofilament, and donkey anti-rabbit IgG secondary antibody conjugated to Alexa 488 (Invitrogen) at 1:500 dilution for antifungal antibodies. To visualize nuclei, sections were then stained with DAPI (4,6- diamino-2-fenilindol) (Merck) and treated with autofluorescence eliminator reagent (Merck). The use of this reagent is important to avoid autofluorescence, since there is lipofuschin in the aging brain. All images were collected and analyzed with a LSM710 confocal laser scanning microscope combined with the upright microscope stand AxioImager.M2 (Zeiss), running Zeiss ZEN 2010 software. The spectral system employed was Quasar + 2 PMTs. Images were deconvoluted using Huygens software (4.2.2 p0) and visualized with Fiji/ImageJ (NIH, Bethesda, MD) software.

## Results

### Fungal proteins are present in CA from AD patients

**Figure 1:**
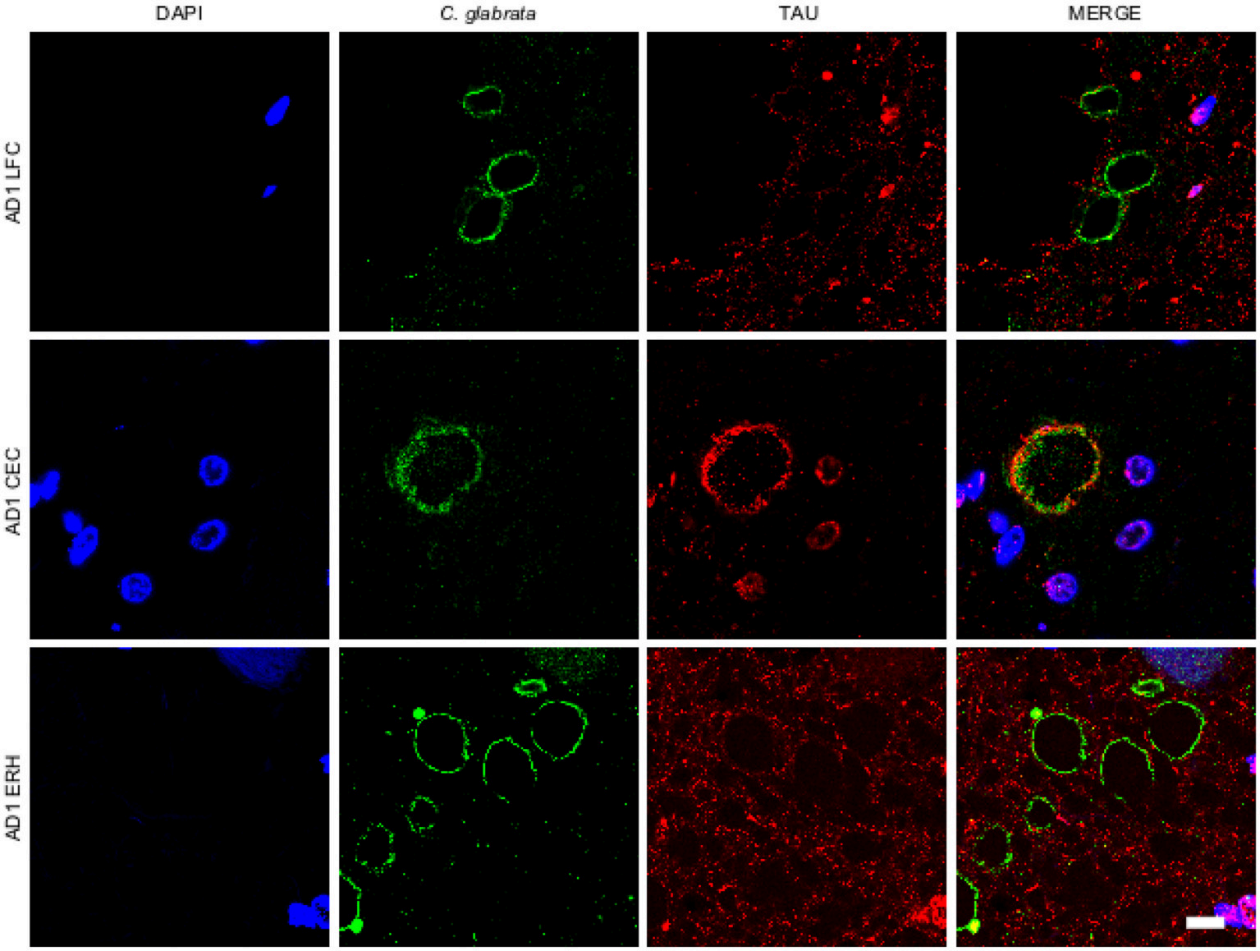
**Corpora amylacea are detected in different regions of the CNS**. Tissue sections (5 μm) from different CNS regions of patient AD1 were tested as follows: lateral frontal cortex (LFC), cerebellar cortex (CEC) and entorhinal cortex/hippocampus (ERH). Immunohistochemistry analysis was carried out by double immunofluorescence staining employing a rabbit polyclonal anti-*C. glabrata* antibody (green) and a mouse monoclonal anti-tau antibody (red). Sections were mounted and observed by confocal microscopy after incubation with the corresponding secondary antibodies, as described in Materials and Methods. Overlapping red and green pixels appear as orange/yellow. DAPI appears in blue. Scale bar: 10 μm.

A variety of cellular proteins constitute part of CA (Sfanos et al., 2009). To assess the presence of fungal proteins in CA, we carried out immunohistochemistry analysis using a specific rabbit polyclonal antibody raised against *C. glabrata*, which does not cross-react with human proteins (Pisa et al., 2015a,b). Initially, we tested this antibody on tissue sections from different CNS regions, including lateral frontal cortex (LFC), cerebellar córtex (CEC) and entorhinal cortex/hippocampus (ERH) from one AD patient. Double immunofluorescence staining was performed using a second antibody that detects tau protein. CA were more abundant in ERH regions than in LFC or CEC (Figure 1). CA from all three CNS regions (LFC, CEC, and ERH) stained positive with the anti-fungal antibody (Figure 1). The external laminar structures and the envelope surrounding the central core of CA were clearly immunoreactive for the *C. glabrata* antibody (green), revealing the presence of fungal proteins in this region (Figure 1). In some instances, the entire envelope was positive, while in other sections only a part of the external envelope was immunoreactive. By contrast, the anti-tau antibody (red) stained only some of the CA, particularly from CEC sections (Figure 1). This result is consistent with previous findings describing tau protein in CA (Singhrao et al., 1993; Day et al., 2015). Of note, not all CA contained tau protein inasmuch as it was undetectable in some CA sections analyzed by confocal microscopy. Thus, although tau can be detected in some CA, it is not an abundant protein.

**Figure 2:**
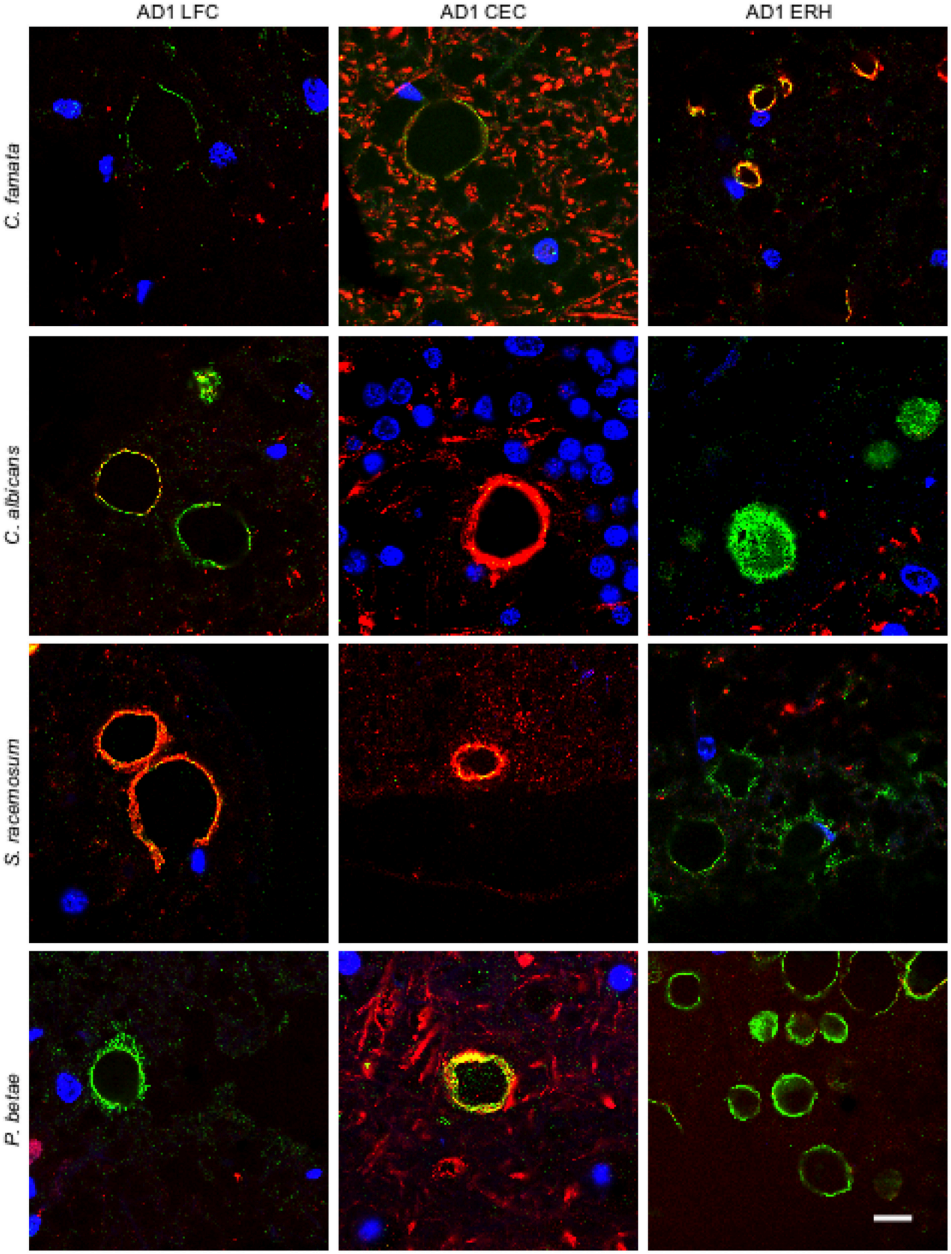
**Immunoreactivity of corpora amylacea against different antifungal antibodies**. Tissue sections analyzed from patient AD1 are indicated in the figure. Immunohistochemistry analysis was carried out using a monoclonal antibody to human neurofilaments (red) and rabbit polyclonal antibodies raised against the following fungi: *C. famata, C. albicans. S. racemosum*, and *P. betae* (green). Sections were mounted and examined by confocal microscopy after incubation with the corresponding secondary antibodies. Orange/yellow corresponds to red and green pixels. DAPI (blue) and scale bar: 10 μm.

To further assess the presence of fungal proteins in CA, tissue sections were immunolabeled with additional antibodies raised against *C. famata, C. albicans. S. racemosum*, and *P. betae* (green) together with a monoclonal antibody against neurofilaments (red). As shown in Figure 2, CA inclusions from the different CNS regions from AD patient 1 immunoreacted with all four antifungal antibodies, further supporting the existence of fungal proteins in CA. None of the antifungal antibodies recognize cellular proteins from neural cells in CNS sections (Pisa et al., 2015a,b). The localization of the immunopositive structures using the additional antibodies was similar to that observed with the *C. glabrata* antibody, strengthening the notion that fungal proteins are present in CA from different CNS regions. By contrast, neurofilament staining was more irregular, with strong immunoreactivity in some CA, and weaker or no staining in other CA inclusions. This was evident in CEC sections double-labeled with the anti-*C. albicans* antibody and in LFC and CEC sections labeled with the anti-*S. racemosum* antibody; in both cases, the neurofilament signal was more intense than the fungal signal, as manifested by the punctate yellow staining in merged images in some of the sections. The fact that neurofilaments are detected in some CA but not in others underscores the concept that the protein composition of CNS CA is nonhomogeneous. This lack of homogeneity can be revealed only by immunohistochemistry and not by proteomic analyses of purified CA.

**Figure 3:**
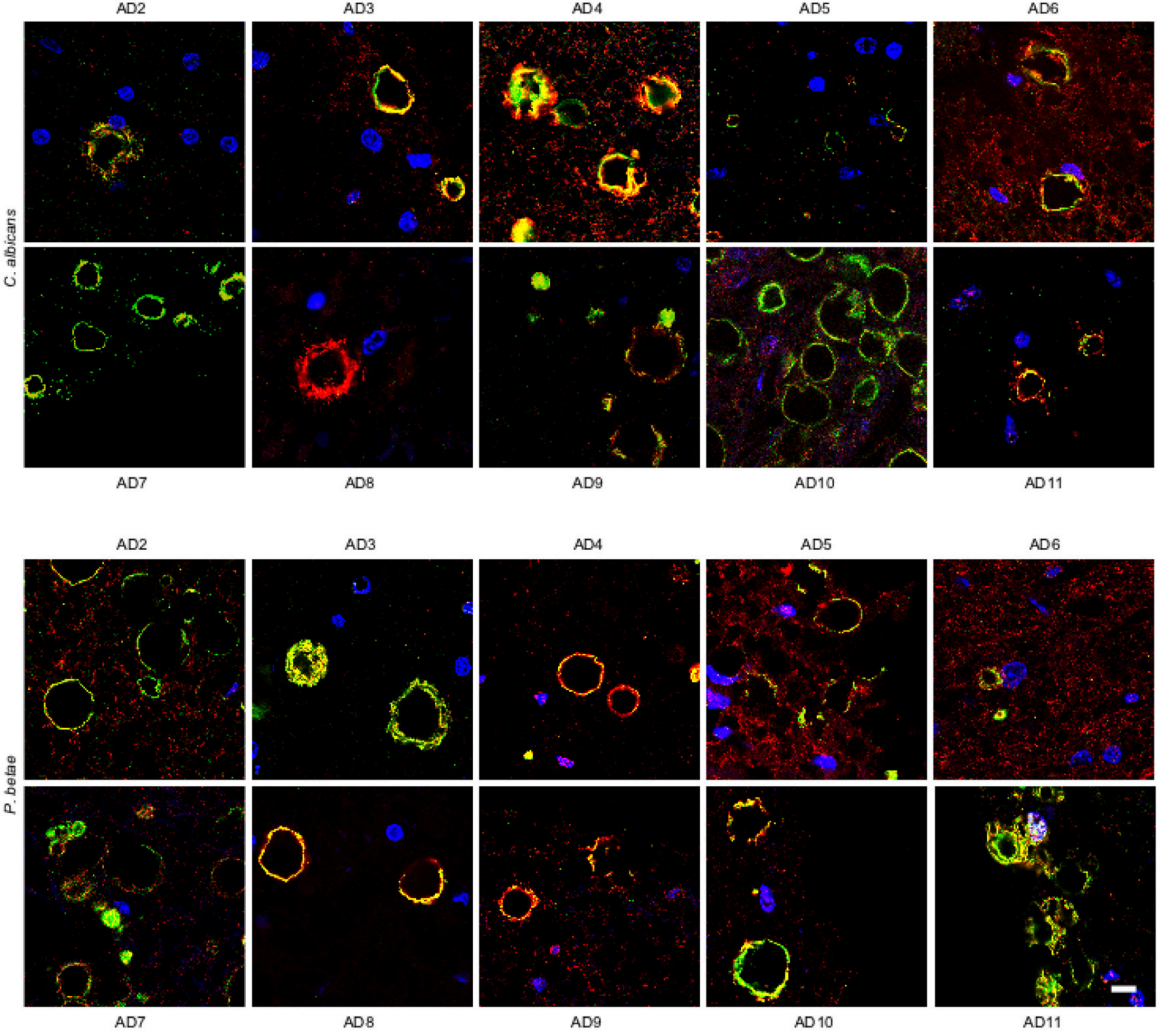
**Detection of fungal proteins in corpora amylacea from different AD patients**. ERH sections from 10 AD patients (AD2-AD11) were incubated with rabbit polyclonal antibodies against *C. albicans* and *P. betae* (green) and a monoclonal antibody against α-tubulin (red). Sections were mounted and examined by confocal microscopy after incubation with the corresponding secondary antibodies. DAPI and scale bar as in Figure 1.

We also analyzed ERH sections from other AD patients (AD2-AD11; Supplementary Table 1). Immunohistochemmistry was performed using anti-*C. albicans* and anti-*P. betae* antibodies (green) and anti-human α-tubulin antibodies were used to mark microtuble structures (red). Of note, the anti-tubulin antibody immunoreacts not only with human cells but also with several eukaryotic species, including fungal cells. Consequently, in the instances where both green and red signals co-localized, it may be because of the presence of fungal tubulin. Fungal proteins were detected in ERH CA inclusions from all 10 additional AD patients (Figure 3). As the antifungal antibodies are polyclonal, they can cross-react with a number of fungal proteins. The positive immunoreactivity with one of these antibodies does not demonstrate that the fungal species present is the same as that employed to raise the antibody. However, since each antibody immunoreacts with different fungal antigens, differences in the immunostaining provides a clue to indicate that the fungal species differ. Accordingly, this technique cannot establish the precise fungal species present in each sample and DNA sequencing would be required (Alonso et al., 2014a,b; Pisa et al., 2015b). The majority of the CA inclusions from different patients immunoreacted with both anti-*C. albicans* and anti-*P. betae* antibodies, although staining for *P. betae* was more robust in patients AD3, AD8, AD10, and AD11 than in the other patients (Figure 3). This finding suggests that the fungal species present in each patient differ. Additionally these results support the use of a panel of anti-fungal antibodies to comprehensively determine the presence or absence of fungal proteins in CA. An important conclusion from this analysis is that the location of fungal proteins in CA inclusions rules out the possibility that fungal infection was due to postmortem contamination since the formation of CA occurs over long time periods (months or even years).

### Analysis of CA from ALS and PD patients

**Figure 4:**
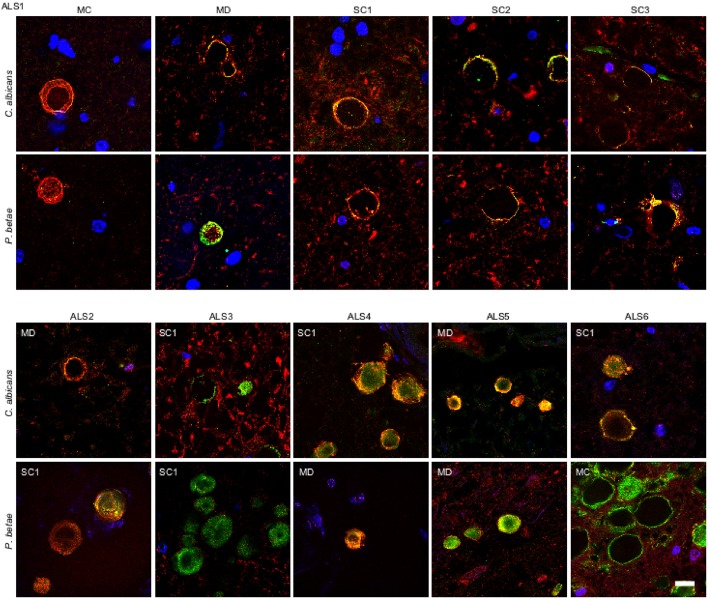
**Fungal proteins in corpora amylacea from ALS patients**. Tissue sections of patient ALS1 were obtained from the following regions: primary motor cortex (MC), medulla (MD) and different levels of the spinal cord (SC1, SC2, and SC3) (upper panels). Different regions of the CNS from five additional ALS patients (ALS2–ALS6) were also analyzed (lower panels). Sections were incubated with rabbit polyclonal antibodies against *C. albicans* and *P. betae* (green) and a monoclonal antibody against α-tubulin (red). Sections were mounted and examined by confocal microscopy after incubation with the corresponding secondary antibodies. DAPI and scale bar as in Figure 1.

Our recent finding of fungal infection in CNS from ALS patients (Alonso et al., 2015), prompted us to test for fungi in CA inclusions in ALS samples. We examined tissue sections from motor cortex (MC), medulla (MD) and different levels of the spinal cord (SC1, SC2, and SC3) of an ALS patient (ALS1) using anti-*C. albicans* and anti-*P. betae* antibodies. Double immunolabeling of the CNS from patient ALS1 with α-tubulin (red) revealed fungal proteins (green) at the periphery of CA inclusions in different regions (Figure 4). Of note, CA inclusions were also detected in different regions of the CNS of this patient, including the spinal cord. The numbers of CA inclusions observed in these samples were, however, lower than those found in AD patients. We also tested tissue sections from different CNS regions of five additional ALS patients (ALS2-ALS6) using the same antibodies. CA inclusions were detected in all ALS patients examined and all were positive for fungal protein. For clarity, only one field with each antibody and only one CNS region for each ALS patient is shown (Figure 4). In general, fungal proteins (green) were detected at the periphery of CA inclusions, but in some instances fungal proteins were observed throughout CA bodies, including the central portion. Additionally, anti-α-tubulin (red), reactivity was in the main detected in association with material present in CA, but in a few instances α-tubulin was detected in the surrounding areas. The observations in ALS tissue indicate that there is a similarity between ALS CA and AD CA (Figures 1, 2), and also further support the concept of CA protein heterogeneity.

**Figure 5:**
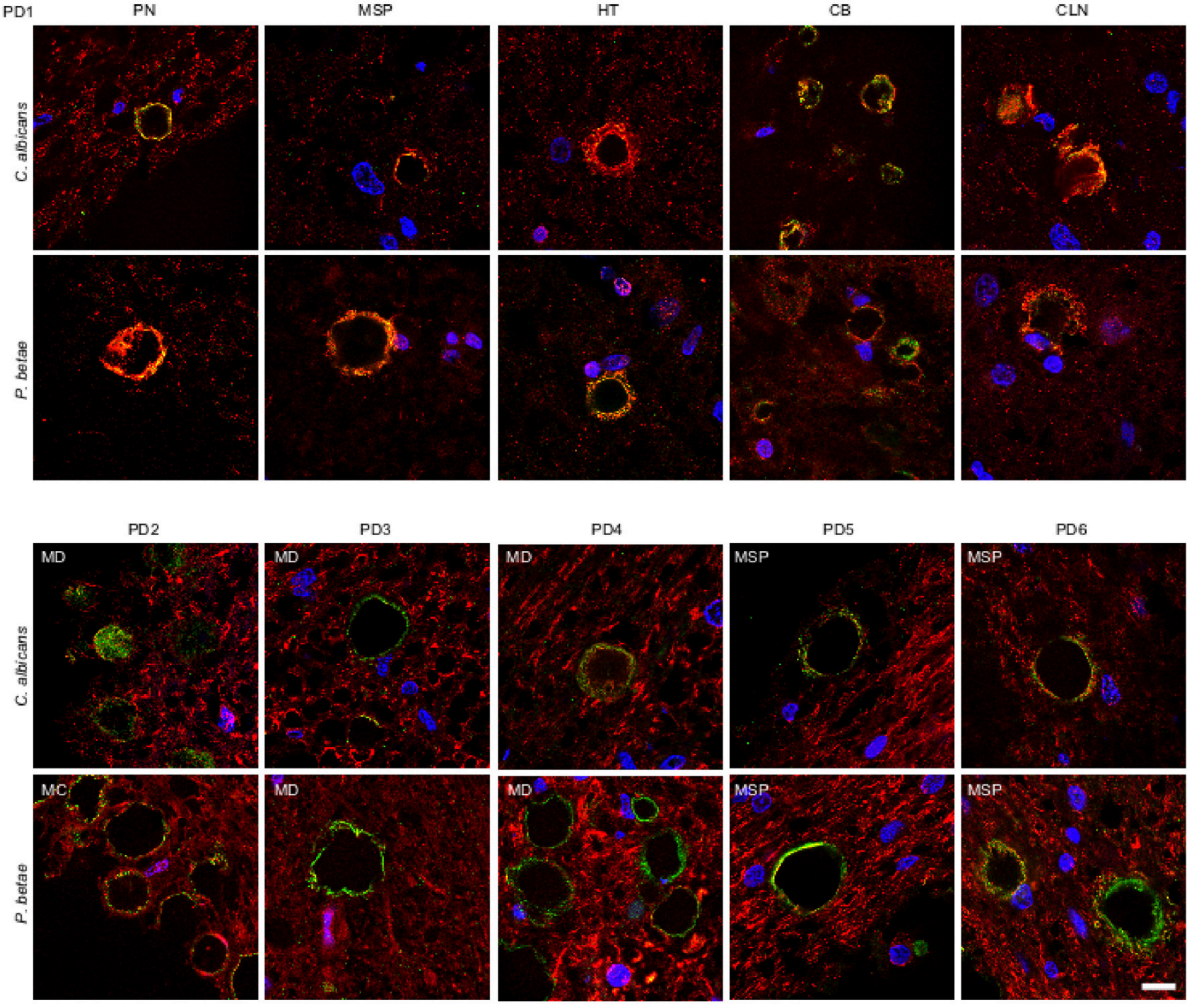
**Detection of fungal proteins in corpora amylacea from PD patients**. Tissue sections of patient PD1 were obtained from the following regions: pons (PN), mesencephalon (MSP), hypothalamus (HT), callosal body (CB), and caudate and lenticular nuclei (CLN) (upper panels). Different regions of the CNS from five additional PD patients (PD2-PD6) were also analyzed (lower panels). Sections were incubated with rabbit polyclonal antibodies against *C. albicans* and *P. betae* (green) and a monoclonal antibody against α-tubulin (red). Sections were mounted and examined by confocal microscopy after incubation with the corresponding secondary antibodies. Overlapping red and green pixels appear as orange/yellow. DAPI and scale bar as in Figure 1.

We also tested for fungal proteins in CA from brain samples of one PD patient. As before, several CNS regions were analyzed using antifungal antibodies (green) and anti-α-tubulin antibodies (red) (Figure 5). The CNS regions examined in this PD patient included pons (PN), mesencephalon (MSP), hypothalamus (HT), callosal body (CB), and caudate and lenticular nuclei (CLN). CA were also detected in this PD patient and were found in all CNS regions analyzed. Furthermore, immunoreactive fungal proteins were prominent in CA bodies and the distribution of the immunolabels was similar to those observed in ALS and AD patients. Similarly to ALS patients, the number of CA inclusions in the different regions examined in the PD patient was much less than in AD patients. Analysis of CNS samples from five additional PD patients using anti-*C. albicans* and anti-*P. betae* antibodies is shown in Figure 5. Once again, CA inclusions were detected in all PD patients analyzed and immunoreactivity to fungal proteins in these CA was revealed with the two antifungal antibodies employed. The location of fungal proteins (green) and the distribution of human α-tubulin were again similar to the pattern observed with CA from ALS patients.

### Study of CA from control individuals

**Figure 6:**
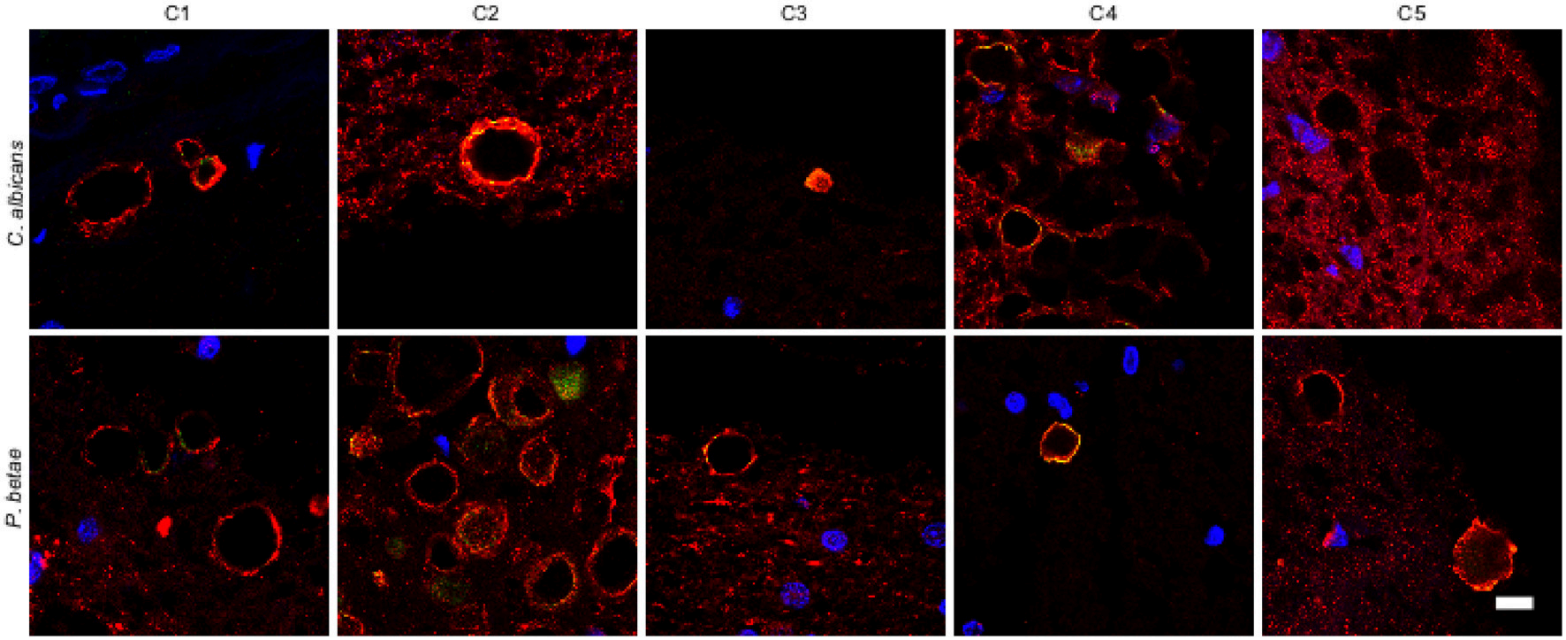
**Analysis of corpora amylacea present in the CNS of control individuals**. Tissue sections from the ERH of five control individuals (C1–C5) were analyzed by immunohistochemistry. Sections were incubated with rabbit polyclonal antibodies against *C. albicans* and *P. betae* (green) and a monoclonal antibody against α-tubulin (red). Sections were mounted and examined by confocal microscopy after incubation with the corresponding secondary antibodies. Overlapping red and green pixels appear as orange/yellow. Note the near absence of activity against the fungal antibodies. DAPI and scale bar as in Figure 1.

**Figure 7:**
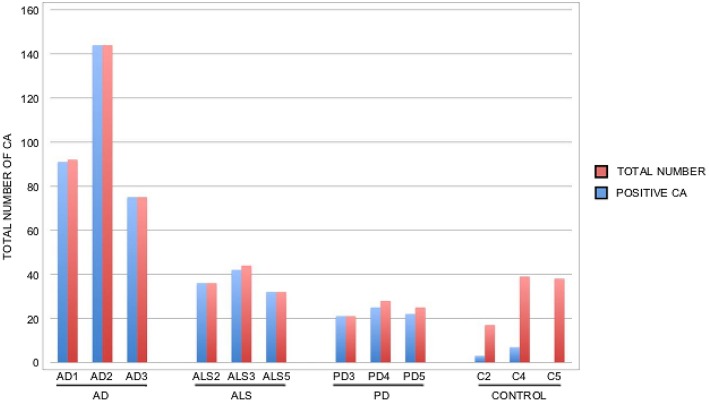
**Quantitation of the number and positiveness of CA from different patients and control subjects**. Brain sections from the patients indicated in the Figure were examined by confocal microscopy and the total number of CA in three different fields was estimated, as well as their staining with anti-*P. betae* antibodies. Wide fields were analyzed using 40x magnification. The sections examined were ERC from AD patients and control subjects, MD from ALS patients, PD3 and PD5 and MC from PD4. Red bar: total number of CA in the three fields; blue bar: positive CA with antifungal antibody.

It is thought that the formation of CA inclusions are related to the aging process, even in healthy subjects (Song et al., 2014). We examined ERH sections from the CNS of five control subjects using the two antifungal antibodies indicated above. In general, CA inclusions were clearly much less abundant than those observed in CNS tissue from patients diagnosed with neurodegenerative diseases. Nevertheless, the CA inclusions in control subjects exhibited a modest immunoreactivity against the two antifungal antibodies employed. For example, CA from control subjects C2 and C3 immunoreacted with the anti-*C. albicans* antibody, while C4 and, to a lesser extent, C3 immunoreacted with the anti-*P. betae* antibody (Figure 6). Similar to CA from neurodegenerative patients, the external perimeter of CA exhibited punctate immunoreactivity when detected; however, the labeling intensity was lower than that observed in AD patients. These findings indicate that in general the amount of fungal proteins in CA from control individuals is much lower than from equivalent neurodegenerative disease patients and in some cases no immunoreactivity is detected. The absence of fungal proteins in some CA might be determined by the particular section examined. Nevertheless, the amount of fungal proteins in control CA inclusions is very low. Alternatively, it is theoretically possible that the fungal proteins, if present, cannot be detected with the antibodies employed in this study. An estimation of the number and positiveness of CA in different patients and control subjects is shown in Figure 7. Certainly, the vast majority of CA from the different patients analyzed is stained with antifungal antibodies, whereas only a very minor portion of CA can be considered as positive in control subjects. On the other hand, in general this quantitation reveals higher numbers of CA in AD patients, particularly from ERC areas where CA are more abundant both in patients and controls. However, one limitation of this quantitation is that these numbers can vary depending on the specific tissue section analyzed. As previously noted by other researchers, the amount of CA is higher close to blood vessels (Nishio et al., 2001).

Collectively, these findings reveal the presence of CA inclusions in several regions of the CNS from patients with neurodegenerative diseases. CA are more abundant in the ERH of AD patients than in other regions but there are higher quantities of CA from ALS and PD samples than in controls. Importantly, fungal proteins are detected in CA from all patients with neurodegenerative diseases tested by means of specific antibodies.

## Discussion

Although great progress has been made in our understanding of the protein composition of CA inclusions (Sfanos et al., 2014), a number of components remain unidentified. Furthermore, the precise origin and potential function of CA inclusions is obscure and subject to speculation (Mrak et al., 1997; Keller, 2006; Song et al., 2014). The proposal that CA originate through neurodegeneration is based on findings showing that several proteins from neural cells can be detected in CA (Singhrao et al., 1993; Selmaj et al., 2008). Along this line, CA are thought to be formed by cellular debris and/or breakdown products from brain cells since cleaved tau protein is found in CA (Day et al., 2015). Another suggestion is that CA arise from a conglomeration of proteins that interact after neuron degeneration and from extravasated blood proteins after breakdown of the hematoencephalic barrier (Meng et al., 2009). Following this idea, CA could be envisaged as aggregated proteins and polyglucans, which together with calcium salts accumulate extracellularly. Thus, some proteins may form insoluble aggregates that are integrated as the amyloid component of CA (Kimura et al., 1998; Vogl et al., 2012). The fact that glial CA inclusions contain heat-shock proteins, heme oxygenase-1 and ubiquitin suggests the existence of oxidative stress, perhaps mediated by advanced glycosylation of cellular proteins (Cisse et al., 1993; Lowe et al., 1993; Iwaki et al., 1996). The possibility that CA are formed from mitocondria inside cells and progressively increase in size leading to cell disruption has also been suggested (Schipper, 2004). Indeed, the expression of heme oxygenase-1 in cultured rat glial cells induces mitochondrial dysfunction and the formation of corpuscles reminiscent of CA (Sahlas et al., 2002; Schipper et al., 2006; Song et al., 2014).

Alternative proposals for the origin of CA center on the idea that microbial infection with concurrent inflammation is an intrinsic component of CA formation (Heinonen et al., 1992; Sfanos et al., 2014). In this vein, the present study demonstrates the existence of fungal proteins in CA from the CNS, but predominantly in patients diagnosed with neurodegenerative diseases since the yield in control individuals is very low. These observations suggest that fungal infection is not necessarily involved in the formation of CA, however, microbial infections and particularly mycoses may enhance and/or trigger the build-up of CA inclusions. Alternatively, if fungal proteins do not participate in CA formation *per se*, it is possible that in tissues infected with fungi, some fungal proteins are conglomerated together with cellular proteins in these inclusions. As CA formation occurs progressively, and the location of these proteins is specific to the CA envelope, the possibility that fungal infection is caused by postmortem contamination is unlikely.

The possibility that CA play a role in the pathology of AD and other diseases has been raised in the literature (Mrak et al., 1997; Keller, 2006; Schipper et al., 2006; Sfanos et al., 2014). If correct, it is conceivable that fungal proteins are also implicated in the severity of symptoms in some of these diseases, mediated by the formation of larger amounts of CA. The possibility that CA, at least in some cases, are formed by mycoses is supported by several lines of evidence. First, CA contain several salts, including calcium phosphate and calcium oxalate, and these salts have also been detected as deposits in fungal infections (Tanaka et al., 1993; Nakagawa et al., 1999; Modem et al., 2006; Rassaei et al., 2009). Second, proteins from neutrophil granules appear in CA and it is well established that fungal infections elicit a neutrophil response (Murthy et al., 1993; Metzler et al., 2011; Cunha et al., 2014). Third, polyglucans form a major component of CA inclusions and this macromolecule is also an integral constituent of the fungal cell wall and is secreted to the external medium (Chaffin et al., 1998; Free, 2013). The finding of keratan sulfate and high mannose glycoconjugates in CA inclusions was initially interpreted as the result of an accumulation of glycoconjugates normally present in the brain tissue matrix through aging (Liu et al., 1987). Nonetheless, it must also be considered that high mannose glycoconjugates are also produced by fungal cells (Chaffin et al., 1998). Therefore, the composition of CA does not discard the possibility that they are related to fungal infection; rather, it is quite feasible that they can originate from mycoses. The findings reported in the present study lend strong support to this possibility. Interestingly, calprotectin exhibits potent anti-*Candida* activity (Sohnle et al., 1996; Okutomi et al., 1998; De Marzo et al., 2007), and this is also the case for defensin (Schroeder et al., 2011), an additional component of CA. Given that amyloid β peptide, which is very abundant in senile plaques, has strong potency against *C. albicans* (Soscia et al., 2010), it is conceivable that an antifungal response occurs in brains from patients diagnosed with some neurodegenerative diseases. Future work aimed at purifying and characterizing the range of polysaccharides present in CA may help to determine whether CA arise as a result of fungal infection.

## Author contributions

DP and RA carried out the experiments. AR managed the human brains and provided the tissue sections. LC designed the experiments and wrote the manuscript. All authors discussed the results obtained and participated in the correction of the manuscript.

### Conflict of interest statement

The authors declare that the research was conducted in the absence of any commercial or financial relationships that could be construed as a potential conflict of interest.
