# Supplementary material for: Corpora Amylacea of Brain Tissue from Neurodegenerative Diseases Are Stained with Specific Antifungal Antibodies
Source: Front Neurosci. 2016 Mar 8;10:86. doi: 10.3389/fnins.2016.00086 (PMC4781869; doi:10.3389/fnins.2016.00086)
Supplement: Supplementary file 1 [file Table1.PDF]

Supplementary Table I.

Description of patients and control subjects analyzed in this work.

SAMPLE	GENDER	AGE
AD1	F	83
AD2	F	80
AD3	F	84
AD4	F	79
AD5	F	81
AD6	M	87
AD7	M	92
AD8	M	81
AD9	F	87
AD10	F	86
AD11	M	62
ALS1	F	56
ALS2	F	69
ALS3	F	NA
ALS4	F	NA
ALS5	M	41
ALS6	F	67
PD1	M	65
PD2	M	77
PD3	M	84
PD4	F	79
PD5	M	82
PD6	M	89
C1	F	77
C2	M	56
C3	F	48
C4	M	63
C5	M	78

NA: not available
F: female
M: male
